# Supplementary material for: Analysis of PPARγ Signaling Activity in Psoriasis
Source: Int J Mol Sci. 2021 Aug 10;22(16):8603. doi: 10.3390/ijms22168603 (PMC8395241; doi:10.3390/ijms22168603)
Supplement: Supplementary file 1 [file ijms-22-08603-s001.zip › Supplemental materials_Analysis of PPARg signaling activity in psoriasis/Pathway models/Models images and html files/PPARG inhibits phenotypes assotiated with psoriasis/189494.html]

growth rate


# Cell Process growth rate

|  |  |
| --- | --- |
| URN | urn:agi-gocellproc:0040009 |
| Total Entities | 0 |
| Connectivity | 4155 |
| Name | growth rate |

---

|  |  |
| --- | --- |
| Pathway | PPARG inhibits phenotypes assotiated with psoriasis |

---

|  |  |
| --- | --- |
| MedScan ID | 4140009 |

---

|  |  |
| --- | --- |
| Alias | rate of growth |
|  | growth velocity |
|  | velocity of growth |
|  | relative growth rates |
|  | growth rates |
|  | rates of growth |
|  | velocities of growth |
|  | relative rate of growth |
|  | growth rate |
|  | relative growth rate |
|  | relative rates of growth |
|  | growth velocities |

---

|  |  |
| --- | --- |
| GO ID | 0040009 |

---
